# Comprehensive Genomic Characterization of Cutaneous Malignant Melanoma Cell Lines Derived from Metastatic Lesions by Whole-Exome Sequencing and SNP Array Profiling

**DOI:** 10.1371/journal.pone.0063597

**Published:** 2013-05-21

**Authors:** Ingrid Cifola, Alessandro Pietrelli, Clarissa Consolandi, Marco Severgnini, Eleonora Mangano, Vincenzo Russo, Gianluca De Bellis, Cristina Battaglia

**Affiliations:** 1 Institute for Biomedical Technologies, National Research Council, Milan, Italy; 2 Cancer Gene Therapy Unit, San Raffaele Scientific Institute, Milan, Italy; 3 Dipartimento di Biotecnologie Mediche e Medicina Traslazionale, Università degli Studi di Milano, Milan, Italy; Virginia Commonwealth University, United States of America

## Abstract

Cutaneous malignant melanoma is the most fatal skin cancer and although improved comprehension of its pathogenic pathways allowed to realize some effective molecular targeted therapies, novel targets and drugs are still needed. Aiming to add genetic information potentially useful for novel targets discovery, we performed an extensive genomic characterization by whole-exome sequencing and SNP array profiling of six cutaneous melanoma cell lines derived from metastatic patients. We obtained a total of 3,325 novel coding single nucleotide variants, including 2,172 non-synonymous variants. We catalogued the coding mutations according to Sanger COSMIC database and to a manually curated list including genes involved in melanoma pathways identified by mining recent literature. Besides confirming the presence of known melanoma driver mutations (*BRAF^V600E^, NRAS^Q61R^*), we identified novel mutated genes involved in signalling pathways crucial for melanoma pathogenesis and already addressed by current targeted therapies (such as MAPK and glutamate pathways). We also identified mutations in four genes (*MUC19*, *PAICS*, *RBMXL1*, *KIF23*) never reported in melanoma, which might deserve further investigations. All data are available to the entire research community in our Melanoma Exome Database (at https://155.253.6.64/MExDB/). In summary, these cell lines are valuable biological tools to improve the genetic comprehension of this complex cancer disease and to study functional relevance of individual mutational events, and these findings could provide insights potentially useful for identification of novel therapeutic targets for cutaneous malignant melanoma.

## Introduction

Cutaneous malignant melanoma is the most fatal form of skin cancer and its worldwide incidence has doubled in the past 20 years. While at early stages it can be surgically treated, metastatic melanoma has a very poor prognosis; furthermore, it is largely refractory to existing therapies and the standard first-line chemotherapy (dacarbazine and high-dose IL-2) has a dramatically low response rate, with severe toxicities [1]. So, melanoma has become an increasingly important public health issue and new therapeutic strategies are urgently needed.

Research studies conducted over the last decade on melanoma molecular pathogenesis improved the elucidation of its key signaling pathways, such as those mediated by Ras/Raf/MEK/ERK (alias MAPK), PI3K/Akt/mTOR, Wnt/b-catenin/APC, MITF and glutamate cascade [2]. The understanding of their broad functional interplays and genetic alteration patterns helped to unravel the complexity and heterogeneity of melanoma and in the meantime provided targets for development of new mutation-driven therapeutic approaches (known as targeted therapy). The best example is represented by the selective BRAF inhibitor vemurafenib, the first FDA-approved targeted drug successfully used on BRAF^V600E^ metastastic melanoma patients [3]. However, due to the rapid acquisition of drug resistance caused by genetic alterations, several additional targeted drugs were developed against MAPK and PI3K/Akt pathways and now they are all used in clinical trials [4], [5]. While primary and acquired resistance are still a problem to be fully understood, studies in preclinical models suggested that combined approaches targeting multiple pathways could be more effective than single-agent therapies for a durable control of metastatic melanoma [6], [7]. Recently, the first combined clinical trial, using BRAF and MEK inhibitors on 162 BRAF^V600^ mutant melanoma patients, announced encouraging results for progression-free survival improvements [8]. Therefore, a comprehensive knowledge of melanoma signalling pathways and mutation patterns could suggest potential targets for novel personalized treatments and combinatorial strategies, to finally improve clinical outcome of cutaneous malignant melanoma patients.

Systematic next-generation sequencing (NGS) approaches such as whole-exome and whole-genome sequencing are powerful tools for cancer mutation discovery providing an unbiased genome-wide screening strategy and allowing the identification of potential causative alterations in unexpected genes [9]. Whole-exome sequencing (WES) enables to sequence all coding regions of the human genome (namely, the exome) and is widely exploited in cancer genomics, as recently implemented by The Cancer Genome Atlas (TCGA) NIH project (http://cancergenome.nih.gov/). Even though, with respect to whole-genome sequencing, WES misses most intronic and regulatory regions, on the other hand it allows to improve sequencing coverage and depth for coding regions, thus providing high sensitivity to discover even low-frequency mutations. So, for basic research and molecular diagnostics applications, WES is currently the best NGS strategy considering extensiveness, time and costs. In the last few years, some WES investigations have been also performed on melanoma cancers [10]–[14]. Overall, these studies evidenced that, having already identified the most recurrent mutations typical of melanoma, now the interest is for low-frequency mutations (“private” mutations), occurring in small groups of cases and providing useful information to potentiate personalized medicine approaches. Anyway, the major challenge remains the discrimination of the few “driver” mutations conferring a selective advantage to tumor cells among the majority of “passenger” mutations with no causative role in cancer progression [14].

Cancer cell lines represent an in vitro model widely accepted to study tumorigenic molecular processes. They have been used to perform molecular and functional analyses useful to understand the role of signalling pathways in cancer initiation and progression, to discriminate potential driver from passenger mutations and to identify novel candidate therapeutic targets [15]. Despite their limitations to reflect human body complexity, tumor cell lines have been also exploited in preclinical studies to screen potential anticancer drugs. The research community agrees that systematic drug screenings on well-characterized cancer cell lines will help to identify genetic predictors of drug sensitivity, useful to assist patient stratification for more personalized cancer treatments. To this purpose, international cancer projects have currently created wide collections of human cancer cell lines from different tumor types, such as the NCI60 panel, the Cancer Cell Line Encyclopedia (CCLE) [16] and the Sanger cell line panel [17], in order to characterize their genetic background and test drug sensitivity, resistance and toxicity.

Here, we provided an extensive genomic characterization for coding mutations and DNA copy number alterations of six melanoma cell lines derived from metastatic lesions of stage IIIc/IV patients by WES and SNP array profiling. We catalogued coding mutations with respect to Sanger COSMIC database and to a manually curated list including genes involved in melanoma pathways identified by querying recent literature. Besides confirming known melanoma driver mutations, we identified novel mutated genes involved in melanoma key signalling cascades. We also identified novel genes never found mutated in melanoma that might represent candidate targets to be further investigated in larger datasets.

## Results and Discussion

To extensively characterize the genetic background of our six cutaneous malignant melanoma cell lines, we performed whole-exome capture by Agilent SureSelect system and ultra-massive sequencing by Illumina technology. Overall, we generated a mean of 74 M raw reads per sample, of which more than 87% successfully mapped to the NCBI human reference genome GRCh37, thus resulting in a mean of 5 Gb of mapped sequences per sample. After duplicate removal, 94.5% of captured target regions were covered by at least one sequencing read (mean coverage), while the mean on-target read depth was 45x (Table S1).

By using GATK tool, we identified more than 128,000 single nucleotide variants (SNVs) per sample, of which over 90% were human known polymorphisms according to NCBI dbSNP132 database (including also Phase I 1000 Genomes Project variants) (Table 1). Thus, we applied a series of progressively stringent filtering steps to reduce this list to a sub-selection of novel and highly confident variations in human coding regions (here termed as HQ-SNVs) (Figure S1). To minimize the false positive rate, we applied a stringent filter on the read depth at the single nucleotide position to be called. In fact, by calculating SNP genotype call concordance between SNP array and WES data in relation to sequencing read depth, we obtained that concordance rate was constantly high for homozygous positions (close to 100% regardless of sequencing depth), while for heterozygous positions it gradually increased as a function of read depth, reaching a 90% plateau with at least 15 reads supporting the interrogated position (Figure S2, Panel A). Thus, we set 15x as the minimum read depth to call SNVs. All samples, but Me05, gave at least 80% on-target coverage at 15x depth (Figure S2, Panel B). Finally, on the whole dataset, we obtained a total of 3,325 novel coding HQ-SNVs, including 1,153 synonymous and 2,172 non-synonymous (both missense and nonsense) variants (Table 1). For each sample we obtained a mean of 362 non-synonymous and 192 synonymous HQ-SNVs. Me01 resulted by far the most mutated among the six melanoma cell lines, while Me05 was the less mutated sample, even if this can partly be due to its lower sequencing yields. Full details for HQ-SNVs detected in each sample are available in our Melanoma Exome Database (MExDB, at https://155.253.6.64/MExDB/) and can be visualized in our GBrowse tool.

**Table 1:** Single nucleotide variants (SNVs) summary.

	Me01	Me02	Me04	Me05	Me08	Me12
**Total SNVs** **detected**	128827	131830	159708	128087	161058	161721
**Novel SNVs** **detected**	11126	8360	10230	6001	9534	7869
**Novel coding** **SNVs detected**	2791	1626	1733	1117	1697	1224
**Novel coding** **HQ-SNVs**	992	563	598	283	563	326
Non-synonymous(NS)	659	385	370	189	369	200
Synonymous (S)	333	178	228	94	194	126
NS/S ratio	1.98	2.16	1.62	2.01	1.90	1.59

Abbreviations: SNVs, single nucleotide variants; HQ-SNVs, high-quality single nucleotide variants.

**Figure 1:**
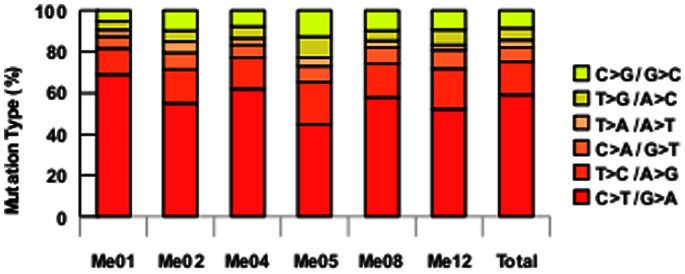
Pattern of nucleotide substitutions. Novel coding single-nucleotide variants were divided and counted according to substitution class in the six melanoma cell lines.

Overall, we calculated a ratio of non-synonymous to synonymous changes (NS/S) of 1.88∶1 (range 1.62–2.16) (Table 1), which was in range with the ratio predicted for non-selected passenger mutations reported by other melanoma WES papers (between 2.0∶1 and 1.9∶1) [11], [12]. According to the known melanoma mutation signature associated with UV light exposure [18], the analysis of nucleotide substitution pattern in all our six melanoma cell lines confirmed that the C>T/G>A transition was the most frequent mutation class (accounting for 45–69% of all substitutions) (Figure 1).

To complete the genomic characterization of these melanoma cell lines, we also assessed their DNA copy number alterations (CNAs) by means of Affymetrix SNP array technology. The six melanoma cell lines showed widely aberrant karyotypes, with large amplifications and deletions involving even entire chromosomes (Figure S3; CNA tracks are also provided by sample in our GBrowse tool). In agreement with the typical melanoma genomic signature, we found that the most recurrent CNAs included amplifications on chrs 1 q, 6 p, 7, 8 q, 17 q and 20, and deletions on chrs 6 q, 10 and 14 q [19].

To characterize our melanoma cell lines respecting already known cancer genes, we compared our non-synonymous SNVs with Sanger COSMIC mutations and our CNA results with the list of COSMIC genes reported as deleted in cancer. Considering overlap on exact genomic positions, we confirmed nine COSMIC mutations (Table 2). The most frequent was the *BRAF^V600E^* mutation occurring in four samples (66%), in a mutually exclusive fashion with the *NRAS^Q61R^* mutation, which we found in one (16%) of the other two cell lines. Me05 was the only melanoma cell line with both *BRAF* and *NRAS* wild-type genes. These are the two oncogenes typically activated by mutation in malignant melanoma, reaching about 60% of cases for BRAF mutations (whose 80% carries the pVal600Glu (V600E)) and 15–30% for NRAS mutations [20]. Also, we found three other mutations already reported in COSMIC for malignant melanoma (XKR6^R162*^, *MADD^S1620F^*, *PKHD1^P1074S^*) and four mutations (in *HYDIN, SPTA1*, *SMC1B* and *MKS1*) described in COSMIC for other tumor types. Full details of characterization of these melanoma cell lines for COSMIC cancer mutations are available in our MExDB. Furthermore, we found 10 COSMIC deleted genes across five melanoma cell lines (Table 2). Me05, with eight deleted genes, resulted the most affected sample. Overall, *CDKN2A* resulted affected by one- or two-copy losses in four samples (66%). Coding for the cell cycle-regulator cyclin-dependent kinase inhibitor 2A, it is the tumor suppressor gene (TSG) most frequently found totally inactivated in up to 70% of melanomas by either homozygous deletion or combination of mutation, one copy-deletion and promoter methylation [19]. In addition, we found deletion of several cancer genes reported in COSMIC for other tumor types, such as *MAP2K4* (described in ovary, lung and breast carcinomas), *APC* (deleted in many different tumor kinds) and all those found in Me05, namely *AXIN1* (reported in stomach and liver adenocarcinomas), *JAK2* and *TET2* (hematopoietic neoplasms), *KIT* (gastrointestinal stromal tumours and hematopoietic neoplasms), *PDGFRA* (gastrointestinal stromal tumours), *RB1* (brain and lung cancers) and *ZRANB1* (ovary carcinoma).

**Table 2:** Confirmed COSMIC mutations and deletions.

	COSMIC genes	
Cell line	Mutated	Deleted
**Me01**	*BRAF, HYDIN, SPTA1, XKR6* *	*CDKN2A* #
**Me02**	*NRAS* Δ, *SMC1B*	*CDKN2A* #, *MAP2K4*
**Me04**	*BRAF* Δ, *MADD, SPTA1*	−
**Me05**	*MKS1*	*AXIN1, CDKN2A, JAK2, KIT, PDGFRA, RB1, TET2, ZRANB1*
**Me08**	*BRAF, PKHD1*	*APC, MAP2K4*
**Me12**	*BRAF*	*CDKN2A* #

In the column “mutated”, we listed COSMIC genes for which we found mutation on the same genomic position as reported in COSMIC database (v58). In the column “deleted”, we listed COSMIC genes for which we confirmed deletion spanning the entire gene locus. Genes recurrent in at least two samples are underlined.

* truncating mutation (stop codon);

Δ homozygous mutation (AF = 1);

# homozygous deletion (two-copy loss).

To characterize the individual genetic background of these cell lines with regards to a more melanoma-focused gene panel, we manually compiled an updated list of 547 melanoma-related genes by combining already existing knowledge and recent literature (File S1; see Methods for details) and we used it to search for known and novel mutations. Then, to highlight particularly impacted molecular processes, we grouped mutated genes according to the signalling cascade or molecular function they belong to. The most relevant classes are reported in Table 3. Overall, Me01 resulted the most mutated sample with 28 mutated genes, while Me12 presented the fewest mutations.

**Table 3:** Melanoma-related genes mutated in the six melanoma cell lines.

	Me01	Me02	Me04	Me05	Me08	Me12	NS/S
**Signalling cascades**							
PI3K/Akt signalling	*PIK3R4*	*PIK3C2G* * §	*PIK3C2G, PREX2* §	*–*	*PIK3CG, PREX2* §	*–*	10/1
MAPK signalling	*MAP3K4, BRAF*	*–*	*BRAF* Δ	*MAPK6*	*MAP2K3, BRAF*	*MAP3K5, BRAF*	8/3
Glutamate signalling	*GRIN2B, GRIN3A, GRM5, PLCB1, PLCB4, PLCE1, PLCZ1*	*GRIN3A*	*GRM1, PLCXD2*	*–*	*–*	*–*	10/13
**Molecular function classes**							
RAS-RAF Ser/Thr kinases	*BRAF*	*NRAS* Δ	*BRAF* Δ	*–*	*BRAF*	*BRAF*	5/0
Protein tyrosine kinases	*FGFR1*	*MET*	*PTK2B*	*PTK7*	*–*	*–*	4/2
Metalloproteinases	*ADAM22* &, *ADAMTS18, MMP24, MMP25*	*ADAMTS9*	*ADAMTS12* *	*–*	*ADAM23, ADAMTS6, ADAMTS9, MMP19*	*–*	10/7
Protein tyrosine phosphatases	*PTPN1, PTPRK*	*PTPN13*	*PTPN13, PTPRF* Δ	*PTPRD* &	*–*	*PTPLA* Δ	7/3
G protein-coupled receptors	*GPR64, GPR101* Δ, *GPR112* Δ, *GPR158, GRM5*	*GPR113* Δ	*GPR151* * Δ, *GRM1*	*GPR112* Δ, *GPR113, GPR133*	*GPR158*	*–*	12/5

* truncating mutation (stop codon);

§ double-mutated in the same sample;

Δ homozygous mutation (AF = 1);

& mutation of one allele together with one-copy deletion of the other one. Genes mutated in at least two samples are underlined. NS/S represents the non-synonymous to synonymous mutation count calculated for the whole molecular pathway or function class.

*BRAF* was the most frequently mutated gene and, together with *NRAS*, they belong to the RAS-RAF Ser/Thr protein kinase family. These two kinases play key roles in melanoma biology since they are upstream activators of both the mitogen-activated protein kinase (MAPK) and the phosphatidyl-inositol-3-phosphate kinase (PI3K)/Akt signalling pathways, promoting uncontrolled tumor growth and survival. The NS/S ratio calculated for this class (5 non-synonymous to 0 synonymous variants) strongly confirmed their driver role in melanoma genesis.

*BRAF* is also included in the MAPK signalling cascade, together with MAP2K/MEK and MAPK/ERK proteins. Recently, two WES papers investigated recurrent mutations in melanoma with a specific focus on *MAP3K* [12] and *MAP2K* families [10]. In our samples, we found 4 other mutated genes belonging to the MAPK cascade, for which we calculated a global NS/S ratio of 2.7∶1 (Table 3). Besides confirming *MAP3K5* with a overall mutation frequency (16%) consistent with that previously described (9–12%) [12], we found a mutation in *MAP3K4* (alias *MEKK4* or *MTK1*), for which loss-of-function mutations have been already reported in 5% of different tumors, but never in melanoma, suggesting a potential tumor suppressive function as also proposed for *MAP3K5* [12], [21]. In addition, we found mutations in *MAP2K3* (alias *MEK3*), a gene never previously reported mutated in COSMIC, but found down-regulated in breast cancer with senescence-promoting functions [22], and in *MAPK6* (alias *ERK3*), occasionally reported mutated in COSMIC for lung, breast and ovary adenocarcinomas and skin squamous cell carcinoma, but never in melanoma, and whose expression resulted strongly induced by BRAF and MEK in melanoma [23]. Since Ras/Raf and MAPK are traditionally targeted by kinase inhibitor drugs currently used in advanced melanoma clinical trials, the knowledge about other potentially involved genes may be useful to suggest novel targets for personalized treatments or combined strategies.

Concerning the PI3K/Akt pathway, we found 4 mutated genes across four samples with a global NS/S ratio of 10∶1 confirming the driver role of this cascade in melanoma genesis (Table 3). According to Berger et al. [13], we confirmed *PREX2* as mutated in two of our melanoma cell lines. Besides confirming one of the mutations already described (the R463C), along with those authors, we observed an atypical mutation pattern, with two and three missense mutations in the same samples, distributed throughout the entire gene length without a classical hot-spot. This genetic feature has been already described for a class of non-canonical oncogenes -such as *ERBB4, FLT3* and *EGFR*- characterized by multiple distributed mutations, each with independent but synergistic effects [24], [25]. Also, we found *PIK3C2G* affected in two melanoma cell lines and double-mutated in one of them (carrying a missense mutation together with a stop codon change). This PI3-kinase gene has been proposed as potential oncogene affected by genomic amplification in ovarian cancer cell lines [26]. Interestingly, another PIK3 gene, the *PIK3CA*, provides the example of a widely studied oncogene of this pathway activated by missense mutations in many tumor types and promoting cell proliferation and invasion via Akt phosphorylation; also, in vitro treatments with PIK3 inhibitors resulted in an effective tumor growth arrest, so suggesting important implications for the development of therapies targeting PIK3-mutated cancers [27]. Moreover, PI3K/Akt inhibitors have been tested in combination with BRAF or MEK inhibitors in melanoma mouse models and human cell lines and showed more promising results than mono-specific targeted approaches [6], [28].

The glutamate pathway is drastically involved in melanoma biology and connected to MAPK and PI3K/Akt cascades. In our dataset, we found 9 mutated genes belonging to this pathway across three samples (Table 3). About ionotropic glutamate receptors (iGluRs), instead of *GRIN2A* already reported for melanoma [11], we found mutations in *GRIN3A* in two samples and *GRIN2B* in one sample. These *GRIN* genes encode different subunits of the N-methyl D-aspartate (NMDA) iGluRs, which were seen critically involved in the regulation of epithelial-mesenchymal transition in renal proximal tubular cells [29]. Especially, *GRIN2B* was proposed as TSG silenced by methylation in several cancer types [30], [31]. Also, treatment with NMDA antagonists demonstrated to inhibit ERK1/2 pathway and reduce tumor growth in lung adenocarcinoma and neuroblastoma mouse models, suggesting the therapeutic potential of inhibiting glutamate pathway for anticancer strategies [32]. About metabotropic glutamate receptors (mGluRs), instead of *GRM3* and *GRM8* already described for melanoma [33], we found mutations in *GRM1* and *GRM5* genes in two distinct samples and we confirmed the involvement of their downstream signalling effector *PLCB4*, according to previous reports [11], together with four other phospholipase C genes. Both *GRM1* and *GRM5* resulted expressed in melanoma cell lines and tissues and induced melanocyte transformation and melanoma formation by ERK signalling activation [34]. Interestingly, *GRM1* and *GRM5* showed to act as independent oncogenes in melanoma mouse models [35]. Concerning their therapeutic potential for melanoma, pharmacological blockade by GRM1 antagonist or glutamate release inhibitor demonstrated to drastically reduce tumor growth rate [36]. Moreover, combined approaches targeting GRM1-mediated glutamate release and multi-Raf kinases in melanoma cell lines showed more promising results than single-agent therapies for a durable control of metastatic disease [7]. Overall, our results confirmed the importance of glutamate signalling in cutaneous malignant melanoma.

Constitutive activation of tyrosine phosphorylation signalling pathways is a classical hallmark of cancer resulting from both activation of protein tyrosine kinase (PTKs) and inactivation of protein tyrosine phosphatases (PTPs). According to Prickett et al. [24], we found *MET*, *PTK2B* and *PTK7* as mutated in three distinct samples (Table 3). Furthermore, we found mutations in six PTP genes across five of our melanoma cell lines. *PTPRD* was mutated in one sample, with an overall frequency similar to that previously reported (12%) [37]; in particular, this gene presented a missense mutation together with complete deletion of the second allele, as already described in melanoma and glioblastoma [37], thus suggesting to be a potential key TSG. *PTPN13* resulted mutated in two samples and both *PTPLA* and *PTPRF* carried an homozygous missense mutation in two distinct melanoma cell lines. Recently, all these genes have been investigated as potential TSGs because down-regulated in lung cancer cell lines; interestingly, *PTPN13* resulted the best candidate TSG since frequently inactivated by deletion or mutation, acting as negative regulator of EGFR and HER2 phosphorylation and activation [38]. In two distinct samples, we found mutations in *PTPRF* and *PTPRK*, which are involved in the regulation of epithelial contacts at adherens junctions and whose inactivation showed to promote colon cancer cell motility [39]. These studies suggest that these genes might play TSG functions also in melanoma pathology and may deserve further investigations.

The metalloproteinase superfamily, including matrix metalloproteinases (MMP), a disintegrin and metalloproteinases (ADAM) and ADAMs with thrombospondin domain (ADAMTS), are proteolytic endopeptidase with several biological functions and widely linked to cancer progression, metastasization and angiogenesis [40]. Recently, they have been extensively screened for coding mutations in melanoma [41], [42]. In our dataset, we found 9 metalloproteinase genes mutated across four samples (Table 3). Besides confirming *ADAMTS18* and *ADAMTS6* [42], we found *ADAMTS9* mutated in two samples and *ADAMTS12* carrying an heterozygous stop codon change in one sample. These two genes have been reported silenced by hypermethylation in colon and esophageal cancers [43], [44]. Furthermore, *ADAMTS12* showed anti-tumorigenic protective functions by blocking ERK signalling activation [45] and its absence in knockout mice resulted in increased angiogenesis and tumor progression [46]. Concerning the ADAM family, *ADAM22* presented a missense mutation in one sample together with complete deletion of the second allele, thus suggesting a potential TSG function. This gene acts in brain as an adhesion molecule inhibiting cell growth by integrin-dependent interactions and was found down-regulated in high-grade gliomas [47]. In addition, *ADAM23*, which we found affected in another sample, was seen often silenced by hypermethylation in many cancer types [48], [49]. On the whole, for all these genes the findings of our exome screening suggest a potential interest as novel candidate melanoma TSGs and indicates the need for further biological investigations.

The G protein-coupled receptors (GPCRs), comprising also the aforementioned mGluRs, are the largest family of cell-surface molecules responsible for regulation of several signalling pathways in many different diseases. In cancer, specific GPCRs are involved in aberrant growth, neo-angiogenesis and metastasis in different tumors and huge efforts are now underway to exploit them and their downstream effectors for development of novel therapeutic strategies [50]. In our dataset, besides mGluRs, we found mutations in 7 other GPCR genes across five samples (Table 3). In addition to *GPR112* reported mutated also by Prickett et al. [33] and carrying a homozygous mutation in two of our samples, we found *GPR113* and *GPR158* each mutated in two samples. While *GPR113* is a poorly studied gene and the only existing reports are those in COSMIC for few stomach and ovary adenocarcinoma cases, *GPR158* has been already reported in COSMIC for a wide range of primary tumors and one skin malignant melanoma, and was found silenced by hypermethylation in esophageal squamous carcinoma cell lines [51]. Furthermore, one of our cell lines presented a homozygous truncating mutation in *GPR151*. For this gene, COSMIC recorded two missense mutations in pancreas and ovary tumors. Interestingly, *GPR151* (alias *GALR4*) encodes a galanin receptor and galanin signalling has been found involved in several diseases and cancer, offering interesting perspectives for receptor-targeting therapeutic approaches [52]; linked to MAPK/ERK pathway, galanin signalling showed to play distinct effects in different tumor types [53], [54]. These evidences supported a role for galanin in tumor biology and proposed its receptors as potential targets for novel therapeutic interventions with selective ligands or receptor antagonists.

Besides the most represented classes here discussed, we also found mutated genes belonging to other well-known molecular processes (complete list in our MExDB). For example, Me01 presented a missense potentially deleterious mutation in the *BRCA2* gene, involved in DNA repair and genome integrity maintenance and already reported mutated in melanoma [10]; this might, at least partly, explain the high number of mutations we exceptionally found in this cell line. Moreover, we found mutations in two ligands (*NRG1* and *NRG3*) of the tyrosine kinase EGF-receptor *ERBB4*, which was recently proposed as novel mutated oncogene for melanoma [24] and was seen involved in glutamate signaling modulation [55], thus strengthening the importance to have a global view of the pathway interactions active in melanoma biology.

Additionally, using our curated melanoma-related list, we characterized our cell lines for genes affected by two-copy losses (or homozygous deletions, HD), an approach traditionally used to identify TSGs completely inactivated in cancer cells. Besides the classical HD of *CDKN2A* in three samples, including also the neighboring *CDKN2B* in two of them, as previously reported [19], we found HD affecting the G protein-receptor gene *GPR21* and the tyrosine kinase *EPHA5* in two distinct cell lines. To our knowledge, this is the first report for *EPHA5* involvement in melanoma, but Prickett et al. [24] already described mutations in melanoma for other *EPH* genes. These genes belong to the ephrin receptor family and contribute to glutamate signaling regulation together with *ERBB4* [56]. Moreover, we found other HDs, all in Me12 sample, involving the *MAP3K7* kinase, the ephrin receptor *EPHA7*, the cyclin-dependent kinases *CCNC* and *CDK19*, and the G protein-coupled receptors *GPR45*, *GPR63*, *GPR6* and *GRPC6A* (this latter already described mutated in melanoma [33]). These results can be visualized by sample in the CN tracks of our GBrowse tool.

Finally, to exploit the potentialities offered by a whole-exome screening, we investigated the presence of mutations in those we called “de novo” genes (i.e. genes not reported in COSMIC v58 nor included in our melanoma-related list). Focusing on multiple occurrences, we found 10 genes mutated in at least two samples (Table 4, full details in our MExDB). These genes showed exclusively non-synonymous variations and some of them presented peculiar mutations or position pattern. For instance, *PAICS* showed the same missense mutation in two samples and in homozygous status in one of them; *MUC19* carried a different truncating mutation in two samples, and in those same samples *MRPL1* presented the same codon affected by two different amino acid changes; *RBMXL1* resulted double-mutated and with the same two missense mutations in two samples; *ZNF66P* was the most recurrent gene mutated in three samples and with a different stop codon change in two of them. By literature mining, we found evidences linking four of these genes to tumorigenesis (see Table 4 for related literature).

**Table 4:** Recurrent de novo mutated genes.

Gene symbol	No. of mutated samples (Sample name)	No. of NS mutations	GO Cellular component	GO Biologicalprocess	Previous reports in cancer [PubMed ID]
*C4orf23*	2 (Me04,Me05)	2	cytoplasm	tRNA processing	
*PAICS*	2 (Me01,Me02)	2	cytoplasm	purine metabolism	ALL [PMID: 15142881], Lung squamous cell carcinoma [PMID: 15246564], Glioblastoma [PMID: 21649900]
*CNST*	2 (Me01,Me12)	2	plasma membrane	Protein transport to membrane	
*KIF23*	2 (Me01,Me08)	2	nucleus	cytokinesis	Glioma [PMID: 21904957], Hepatocellular carcinoma [PMID: 21825042], NSCLC [PMID: 21412013], *KIF20A in melanoma [PMID: 22854760]*
*MRPL1*	2 (Me01,Me08)	2	mitochondrion	translation	
*MUC19*	2 (Me01,Me08)	2	extracellular region	cell adhesion	Salivary gland tumors [PMID: 21072847], Inflammation [PMID: 19533339, 21983784], *MUC1 and MUC4 in lung [PMID: 19318547], breast [PMID: 21277939], prostate [PMID: 16302265], ovarian cancers [PMID: 20697346], melanoma [PMID: 19293191]*
*DCAKD*	2 (Me01,Me04)	2	mitochondrion	coenzyme A biosynthetic process	
*MRPL53*	2 (Me01,Me04)	2	mitochondrion	translation	
*RBMXL1*	2 (Me01,Me04)	4	nucleus	RNA splicing	Gastric cancer [PMID: 19802518], Breast cancer [PMID: 16552754]
*ZNF66P*	3 (Me01,Me04, Me05)	3	nucleus	regulation of transcription	

Literature is indicated by PubMed ID code (PMID). References in italics concern other members of the same gene family involved in cancer and here reported to support a potential interest for the listed mutated genes.

Abbreviations: NS, non-synonymous; GO, Gene Ontology; ALL, acute lymphoblastic leukemia; NSCLC, non-small cell lung cancer.

*MUC19* encodes a gel-forming mucin over-expressed in salivary gland tumors, together with *MUC1* and *MUC4*. Its transcription increases in response to inflammatory cytokines and rare coding variants have been recently associated with inflammatory bowel diseases. This gene belongs to a wide family of glycoproteins (mucins) now emerging as important molecules involved in cell adhesion, polarity disruption and growth pathways activation in inflammation and cancer, and already exploited as highly attractive therapeutic targets for cancer vaccines, antibodies and drug inhibitors [57]. The most prominent genes, *MUC1* and *MUC4*, showed aberrant expression in many tumors, directly connected to invasive and metastatic properties. Little is known about mucins in melanoma, where few papers reported the over-expression of *MUC4* with anti-apoptotic activity conferring chemotherapy resistance. Interestingly, *MUC4* is one of the novel candidate melanoma genes recently identified by whole-genome sequencing [13].

*PAICS*, coding for a purine biosynthesis enzyme, was found expressed in acute lymphoblastic leukemia; currently, this pathway is targeted by many antileukemic agents [58]. In solid cancers, *PAICS* was found over-expressed in lung squamous cell carcinoma, probably reflecting the high proliferation rate of this tumor, and showed a general association with glioblastoma progression-free survival. Interestingly, by querying Oncomine database, we found *PAICS* expression significantly up-regulated in melanoma versus normal samples in Haqq melanoma dataset (p-value = 0.001; https://www.oncomine.com/).

*RBMXL1*, encoding a RNA binding protein involved in splicing process, was down-regulated by miR-421 in gastric cancer cells and its reconstituted expression reduced tumor proliferation. Accordingly, the expression of some *RBMX* genes was found associated with that of pro-apoptotic genes in breast cancer, thus supporting a tumor suppressive role for *RBMXL1*.

*KIF23*, alias *MKLP1*, encodes the mitotic kinesin-like protein 1 and participates to cytokinesis and chromosome segregation during cell cycle. Its increased expression in many different cancer types promoted tumor cell proliferation, suggesting its potential interest as prognostic biomarker and therapeutic target. Recently, another member of this gene family, the *KIF20A*, was proposed as promising target for immunotherapy in advanced melanoma. Taken as a whole, these evidences supported the involvement of these four genes in cancer and suggested their potential interest also for melanoma pathology, where further investigations in larger datasets will be required.

### Conclusions

Here, we performed a comprehensive genomic characterization of six cutaneous malignant melanoma cell lines from metastatic patients that represent precious research tools to better understand melanoma pathways and their functional interplays. The melanoma cell lines here described presented chromosomal karyotypes and mutational profiles well resembling those reported for primary tumors. It is noteworthy that, although the small size of our sample set, in these six melanoma cell lines we detected even some low-frequency mutations only recently discovered in large panels of melanoma tissues, such as *PREX2* identified in 14% of a cohort of 107 metastatic melanomas [13]. We provide full details about characterization of these melanoma cell lines in our MExDB and GBrowse tool, as a publicly available resource for the entire melanoma research community. We are currently planning to expand the molecular characterization of these cells by RNA-seq and promoter methylation analysis, as well as to perform functional assays to ultimately validate the biological role of some potentially interesting mutated genes we identified here. Such results will be included in future works, with the final aim to provide a full molecular characterization of these melanoma cell lines. Although all cancer cell lines are a simplified model of primary tumors and their major limitations remain the impossibility to study the role of tumor microenvironment as well as the pharmacokinetics effects exerted by human body on administered drugs, we propose these six melanoma cell lines as useful tools for basic research and preclinical applications. They may add useful information for development of novel mutation-driven therapeutic approaches and design of combined strategies targeting interconnected pathways, an option which already promised to increase success rate for melanoma therapy. Finally, the opportunity to test drug sensitivity on well-characterized melanoma cells may help to monitor the association between specific mutations and individual drug resistance or toxicity, thus allowing to define alternative treatments. These are the basis to achieve more accurate personalized targeted therapies aimed to maximize treatment efficacy while reducing side effects and resistance, to finally improve survival and quality of life of individuals affected by cutaneous malignant melanoma.

## Materials and Methods

### Melanoma Cell Lines

Cancer cell lines were established in our laboratory from surgical tissue samples of metastatic melanoma patients (Table S2), after written informed consent, as described in Villablanca et al. [59]. All research activities involving human subjects and derived specimens were approved by the Institutional Ethics Committee of Istituto Scientifico San Raffaele (Milan, Italy) and were conducted according to the Declaration of Helsinki. For internal uses, the six melanoma cell lines were labeled with a progressive number code replacing their original sample names reported in Villablanca et al. [59]: Me01 (CIP-5), Me02 (M3M005), Me04 (M3M001), Me05 (MR245), Me08 (Ost) and Me12 (MR268). For additional information see File S2.

### Whole-genome Copy Number Alteration Analysis by Affymetrix SNP Array

Genome-wide analysis of DNA copy number alterations (CNAs) was performed using the GeneChip® Human Mapping 250K SNP Array platform (Affymetrix, Santa Clara, CA, USA) and Partek Genomics Suite software (version 6.5; Partek Inc., St Louis, MO, USA) (full details in File S2).

### Whole-exome Capture and Sequencing

Exome capture was performed using the Agilent SureSelect^XT^ Human All Exon 50 Mb kit (Agilent Technologies, Santa Clara, CA, USA). Ultra-massive sequencing was performed on the Illumina GAIIx instrument, in a paired-end 76-cycle run. Illumina SCS and CASAVA software were used for raw data processing and fastq file generation.

### Whole-exome Sequencing Data Analysis

Whole-exome sequencing (WES) paired-end reads were mapped to the NCBI human reference genome GRCh37 build using MAQ aligner (http://maq.sourceforge.net/). We focused our attention on single nucleotide variants (SNVs), without addressing further investigations to small insertions/deletions (indels) or other possible structural variations. After duplicate removal, GATK software was used for read quality score recalibration, local realignment around indels and SNV calling. Then, to create a sub-selection of novel coding high-quality SNVs (HQ-SNVs) for further analyses, a series of filtering options was applied, including minimum read depth of 15x, SNV call quality score above 150 and non-reference allele frequency above 20% (Figure S1). Moreover, SNVs already present in NCBI dbSNP132 (including also Phase I 1000 Genomes Project variants), located in non-coding regions or mapping on HLA loci or homologous repeated genes were filtered out, as well as SNVs overlapping to variants already found in our in-house WES projects database. On the basis of the ratio calculated by GATK between non-reference and reference reads (termed “allele frequency” (AF)), SNVs were classified as heterozygous (AF = 0.5) or homozygous (AF = 1) variants. Finally, SIFT tool was used to predict potential protein-function damage for non-synonymous missense HQ-SNVs (http://sift.jcvi.org/). Complete details are available in File S2.

### Concordance with SNP Array Genotype Calls

To assess reliability and sensitivity of our WES data for SNV detection, genotype calls generated from WES and Affymetrix SNP array for the six samples were compared and their concordance was evaluated in relation to sequencing read depth (see File S2 for complete description). From this analysis we defined the minimum read depth for SNV calling.

### Bioinformatics Analysis

#### COSMIC gene list

SNV and CNA data were compared with Sanger Catalogue Of Somatic Mutations In Cancer (COSMIC database v58, ftp://ftp.sanger.ac.uk/pub/CGP/cosmic/). Mutation and deletion overlaps were evaluated in order to characterize our samples respecting already known cancer genes.

#### Manually curated list of melanoma-related genes

We manually compiled a comprehensive and updated list of melanoma-related genes by combining genes already known according to traditional knowledge and those proposed as novel candidate genes by recently published literature and extending the list so to include all the members belonging to those given gene families. The final list comprised 30 gene families from the canonical KEGG Melanoma pathway (map05218) and other 26 gene families derived from recent melanoma WES or targeted sequencing papers [10]–[13], [24], [33], [37], [41], [42], [60], for a total of 547 potentially melanoma-related genes (File S1). This list was used to extensively screen our melanoma cell lines for known and novel mutations; then, to highlight particularly impacted molecular processes, mutated genes were grouped according to the signaling cascade or molecular function they belong to.

### Web Resources

All the HQ-SNVs found in each melanoma cell line were collected into our SQLite-based Melanoma Exome Database (MExDB), publicly available at https://155.253.6.64/MExDB/. Moreover, a GBrowse genome viewer is available at http://155.253.6.64/cgi-bin/gb2/gbrowse/melanoma_exome_agilent/to browse throughout chromosomes and samples, for visualizing read alignments, SNV positions and read depth and Partek CNA regions.

## Supporting Information

Figure S1 **Flowchart of WES data analysis pipeline.** The flowchart illustrates the analytical steps carried out to process and analyze WES data, from reads alignment to single nucleotide variant (SNV) calling by GATK and subsequent filtering to obtain a sub-selection of high-quality SNVs (HQ-SNVs). To store, manage and share data produced, a public database (Melanoma Exome Database, MExDB) and a GBrowse genome viewer tool were implemented as freely available resources for the entire research community.
(PDF)

Figure S2 **Genotype call concordance between WES and SNP array data and on-target coverage by sequencing read depth.** In Panel A, SNP call concordance between WES and SNP array data on the whole cell line panel was calculated in relation to sequencing read depth and separately plotted for homozygous reference (AF = 0, blue line), homozygous non-reference (AF = 1, green line) and heterozygous variations (AF = 0.5, red line). Finally, we chose to set at 15x the minimum read depth for SNV calling. In Panel B, for each melanoma cell line, mean coverage on captured target regions was plotted in relation to sequencing read depth. At 15x depth threshold, all samples but Me05 gave at least 80% on-target coverage.
(PNG)

Figure S3 **Whole-genome copy number alteration (CNA) profiles of the six cutaneous malignant melanoma cell lines.** Analysis was performed using Partek Genomics Suite (v6.5) and comparing each melanoma cell line to a normal reference pool. Along each chromosome (from 1 to 22), copy number alteration (CNA) regions are reported by sample (samples are ordered from left to right, from Me01 to Me12). Color code is used to distinguish amplifications (red tracks, for regions with CN value above 2.3) and deletions (blue tracks, for regions with CN value less than 1.3, including both one-copy and two-copy losses). All these CNA regions can be visualized in detail in our GBrowse tool.
(JPEG)

Table S1 **Whole-exome sequencing (WES) results.**
(PDF)

Table S2 **Characteristics of cutaneous malignant melanoma patients and derived cell lines.**
(PDF)

File S1 **List of melanoma-related genes and gene families.** A comprehensive and updated list of melanoma-related genes was created by combining genes and gene families derived from canonical KEGG Melanoma Pathway (map05218) to those proposed by recent melanoma WES or targeted sequencing papers (see File S2 for literature). Finally, the list comprised 56 melanoma-related gene families, for a total of 547 genes. Gene families were grouped according to signalling cascade or molecular function they belong to, in order to have a functional view of mutated genes and highlight particularly impacted molecular processes.
(XLS)

File S2 **Supplementary methods (extended version).**
(PDF)
