# Supplementary material for: Comprehensive Genomic Characterization of Cutaneous Malignant Melanoma Cell Lines Derived from Metastatic Lesions by Whole-Exome Sequencing and SNP Array Profiling
Source: PLoS One. 2013 May 21;8(5):e63597. doi: 10.1371/journal.pone.0063597 (PMC3660556; doi:10.1371/journal.pone.0063597)
Supplement: Figure S1 — Flowchart of WES data analysis pipeline. The flowchart illustrates the analytical steps carried out to process and analyze WES data, from reads alignment to single nucleotide variant (SNV) calling by GATK and subsequent filtering to obtain a sub-selection of high-quality SNVs (HQ-SNVs). To store, manage and share data produced, a public database (Melanoma Exome Database, MExDB) and a GBrowse genome viewer tool were implemented as freely available resources for the entire research community. (PDF) [file pone.0063597.s001.pdf]

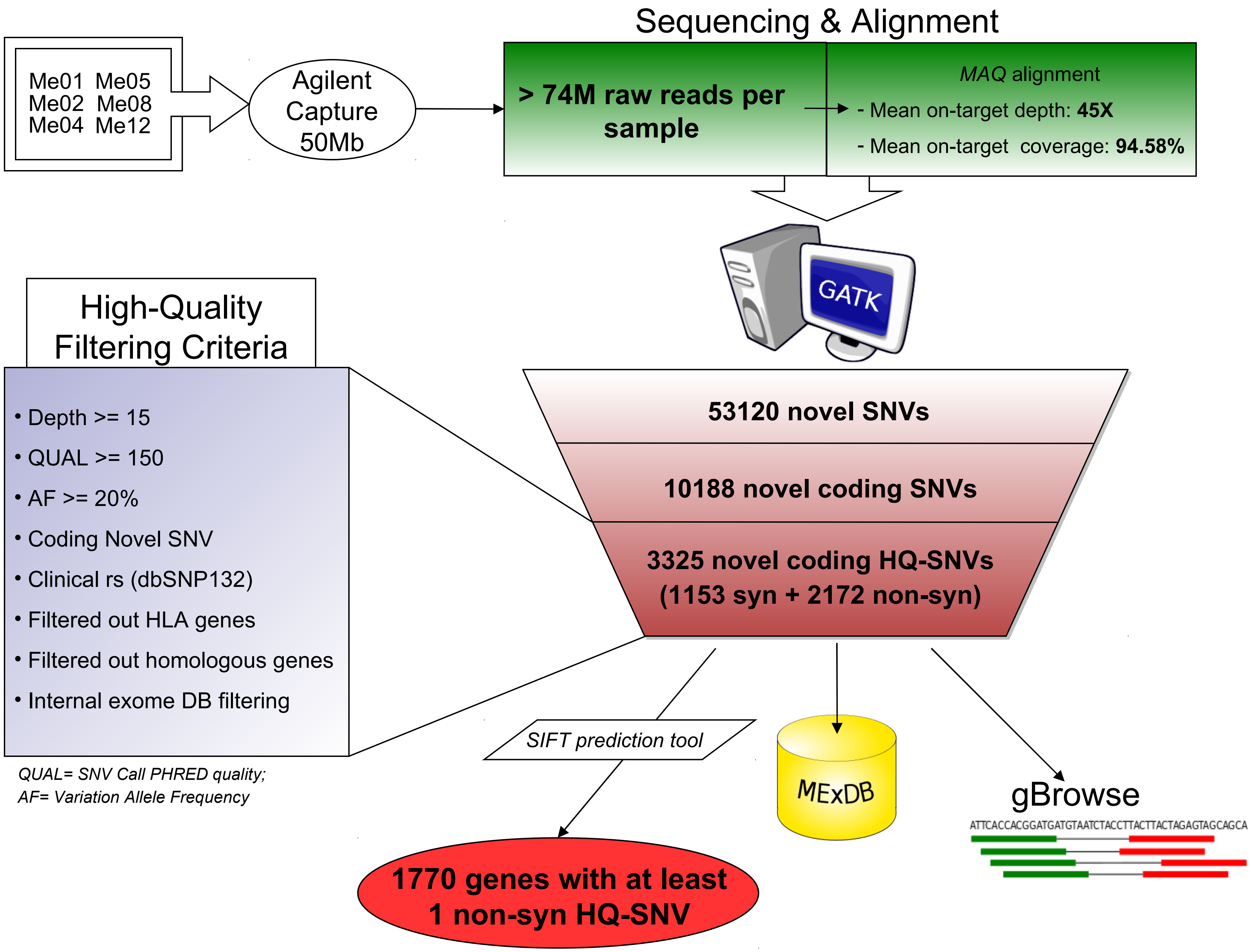

Me01 Me05
Me02 Me08
Me04 Me12
Agilent Capture 50Mb
Sequencing & Alignment
> 74M raw reads per sample
MAQ alignment
- Mean on-target depth: 45X
- Mean on-target coverage: 94.58%
GATK
High-Quality Filtering Criteria
• Depth >= 15
• QUAL >= 150
• AF >= 20%
• Coding Novel SNV
• Clinical rs (dbSNP132)
• Filtered out HLA genes
• Filtered out homologous genes
• Internal exome DB filtering
QUAL= SNV Call PHRED quality;
AF= Variation Allele Frequency
53120 novel SNVs
10188 novel coding SNVs
3325 novel coding HQ-SNVs
(1153 syn + 2172 non-syn)
SIFT prediction tool
MExDB
gBrowse
ATTCACCACGGATGATGTAATCTACCTTACTTACTAGAGTAGCAGCA
1770 genes with at least 1 non-syn HQ-SNV
