# Supplementary figures and images for: Comprehensive Genomic Characterization of Cutaneous Malignant Melanoma Cell Lines Derived from Metastatic Lesions by Whole-Exome Sequencing and SNP Array Profiling
Source: PLoS One. 2013 May 21;8(5):e63597. doi: 10.1371/journal.pone.0063597 (PMC3660556; doi:10.1371/journal.pone.0063597)

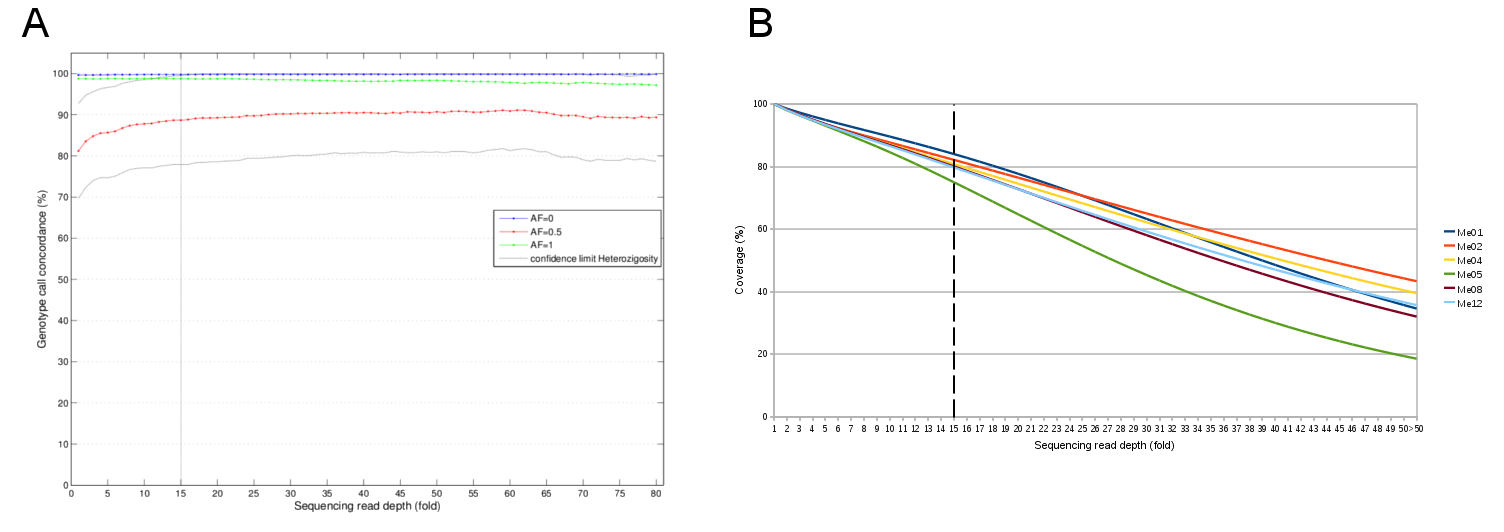

Supplement: Figure S2 — Genotype call concordance between WES and SNP array data and on-target coverage by sequencing read depth. In Panel A, SNP call concordance between WES and SNP array data on the whole cell line panel was calculated in relation to sequencing read depth and separately plotted for homozygous reference (AF = 0, blue line), homozygous non-reference (AF = 1, green line) and heterozygous variations (AF = 0.5, red line). Finally, we chose to set at 15x the minimum read depth for SNV calling. In Panel B, for each melanoma cell line, mean coverage on captured target regions was plotted in relation to sequencing read depth. At 15x depth threshold, all samples but Me05 gave at least 80% on-target coverage. (PNG) [file pone.0063597.s002.png]

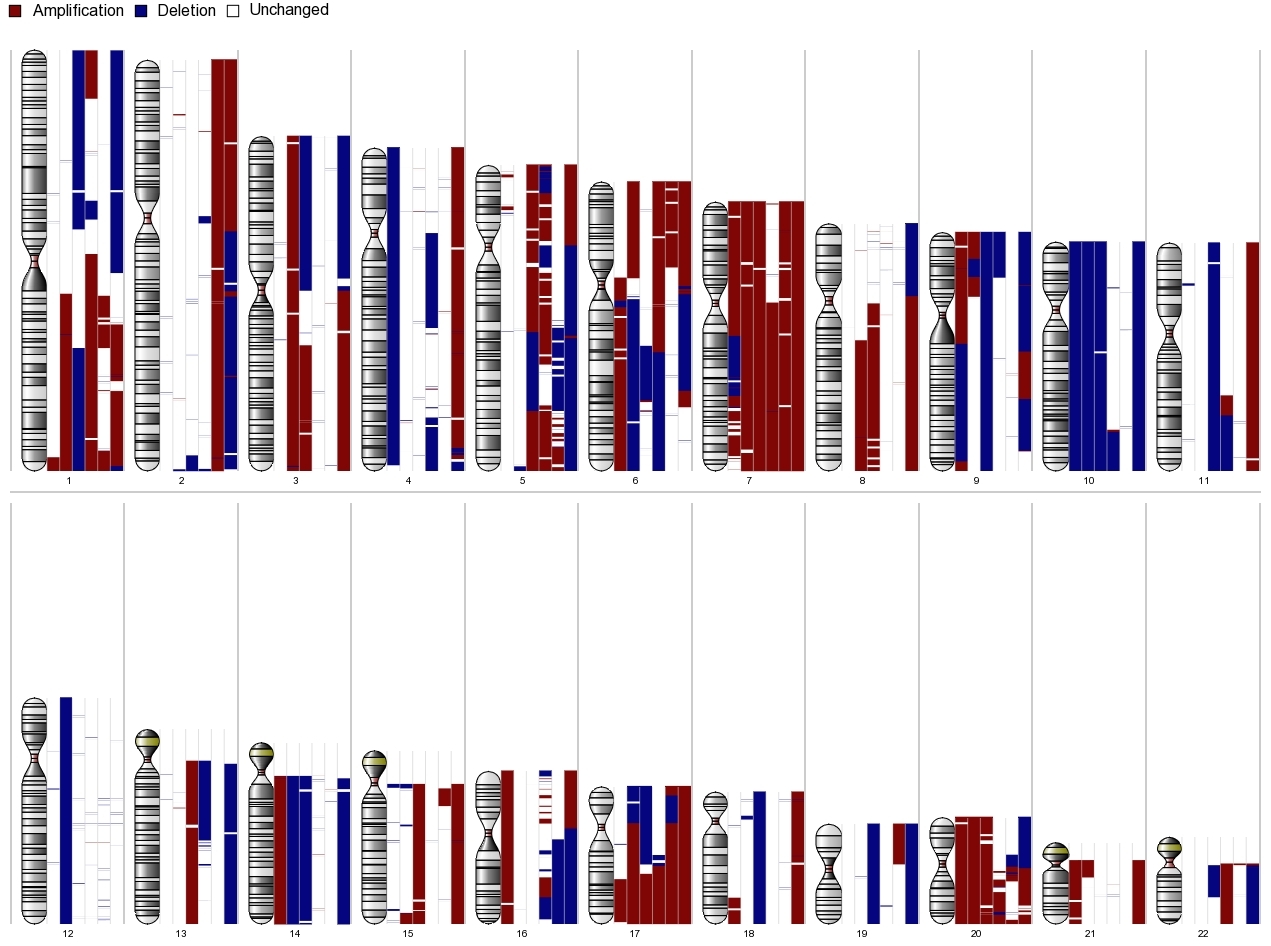

Supplement: Figure S3 — Whole-genome copy number alteration (CNA) profiles of the six cutaneous malignant melanoma cell lines. Analysis was performed using Partek Genomics Suite (v6.5) and comparing each melanoma cell line to a normal reference pool. Along each chromosome (from 1 to 22), copy number alteration (CNA) regions are reported by sample (samples are ordered from left to right, from Me01 to Me12). Color code is used to distinguish amplifications (red tracks, for regions with CN value above 2.3) and deletions (blue tracks, for regions with CN value less than 1.3, including both one-copy and two-copy losses). All these CNA regions can be visualized in detail in our GBrowse tool. (JPEG) [file pone.0063597.s003.jpeg]
