# Supplementary material for: Comprehensive Genomic Characterization of Cutaneous Malignant Melanoma Cell Lines Derived from Metastatic Lesions by Whole-Exome Sequencing and SNP Array Profiling
Source: PLoS One. 2013 May 21;8(5):e63597. doi: 10.1371/journal.pone.0063597 (PMC3660556; doi:10.1371/journal.pone.0063597)
Supplement: Table S1 — Whole-exome sequencing (WES) results. (PDF) [file pone.0063597.s004.pdf]

Supplementary Table S1: Whole-exome sequencing (WES) results.

Exome capture and sequencing results are reported for each sample throughout all the analytical steps, starting from reads generation and mapping counts to depth and coverage statistics on captured target regions.

	Cell line					
	Me01	Me02	Me04	Me05	Me08	Me12
Number of raw reads	69721034	91072538	84459524	50671560	74518054	83755682
Number of mapped reads (% of mapped/total reads)	61114446 (87.66)	80605458 (88.51)	74493737 (88.20)	44052388 (86.94)	65200442 (87.50)	73408916 (87.50)
Mean read depth on target regions (fold)	44.54	54.37	50.78	32.71	42.78	46.38
Mean coverage on target regions (%)	94.96	94.25	94.45	94.66	94.70	94.44
Coverage at ≥ 15x depth (%)	83.32	82.47	80.01	74.05	80.01	79.69
