# Supplementary material for: Comprehensive Genomic Characterization of Cutaneous Malignant Melanoma Cell Lines Derived from Metastatic Lesions by Whole-Exome Sequencing and SNP Array Profiling
Source: PLoS One. 2013 May 21;8(5):e63597. doi: 10.1371/journal.pone.0063597 (PMC3660556; doi:10.1371/journal.pone.0063597)
Supplement: Table S2 — Characteristics of cutaneous malignant melanoma patients and derived cell lines. (PDF) [file pone.0063597.s005.pdf]

Supplementary Table S2: Characteristics of cutaneous malignant melanoma patients and derived cell lines.

Melanoma cell lines were derived from surgical specimens of metastatic lesions of six cutaneous malignant melanoma patients, as described in Villablanca et al. (2010). For internal uses, melanoma cell lines were labeled with a progressive number code replacing their original sample names reported in Villablanca et al. (2010). For each case, gender, disease familiarity, stage of melanoma disease and metastasis site from which tumor cell line was derived are reported.

Internal number code	*Original sample name*	*Gender*	*Familiarity*	*Disease stage*	*Site of origin of tumor cell line*
Me01	CIP-5	M	No	IV	Muscular metastasis
Me02	M3M005	F	No	IIIc	Subcutaneous metastasis
Me04	M3M001	F	No	IV	Muscular metastasis
Me05	MR245	M	No	IV	Lymph node metastasis
Me08	Ost	M	No	IV	Subcutaneous metastasis
Me12	MR268	M	No	IV	Lung metastasis
