# Supplementary material for: Comprehensive Genomic Characterization of Cutaneous Malignant Melanoma Cell Lines Derived from Metastatic Lesions by Whole-Exome Sequencing and SNP Array Profiling
Source: PLoS One. 2013 May 21;8(5):e63597. doi: 10.1371/journal.pone.0063597 (PMC3660556; doi:10.1371/journal.pone.0063597)
Supplement: File S2 — Supplementary methods (extended version). (PDF) [file pone.0063597.s007.pdf]

## SUPPLEMENTARY METHODS – Full version

### Melanoma cell lines

Cancer cell lines were obtained in our laboratory from surgical tissue samples of metastatic melanoma patients (Supp Table S2), after written informed consent, as described in Villablanca et al. (2010). All research activities involving human subjects and derived specimens were approved by the Institutional Ethics Committee of Istituto Scientifico San Raffaele (Milan, Italy) and were conducted according to the Declaration of Helsinki. For internal uses, the six melanoma cell lines were labeled with a progressive number code replacing their original sample names reported in Villablanca et al. (2010): Me01 (CIP-5), Me02 (M3M005), Me04 (M3M001), Me05 (MR245), Me08 (Ost) and Me12 (MR268). All the cell lines were cultured in Iscove's modified Dulbecco's medium (Sigma-Aldrich, Schnelldorf, Germany) supplemented with 10% heat-inactivated fetal bovine serum (FBS, HyClone Laboratories, Logan, UT, USA), 1% penicillin/streptomycin (Sigma-Aldrich) and 2mM L-Glutamine (Sigma-Aldrich), and propagated in T25 flasks at 37°C in 5% $CO_2$. When at confluence, cells were detached with 1x Trypsin-EDTA solution (Sigma-Aldrich), washed twice in Dulbecco's Phosphate Buffered Saline (PBS, Sigma-Aldrich), pelleted by centrifugation and snap-frozen in dry ice. Pellets were stored at -80°C until used for DNA extraction.

### Sample preparation for Affymetrix GeneChip® SNP array

Genomic DNA samples were extracted using the DNeasy Mini kit (Qiagen, Hilden, Germany), according to manufacturer's instructions, and eluted in 200 ul Qiagen EB Buffer. After extraction, DNA from Me01 cells was cleaned up by using the OneStep™ PCR Inhibitor Removal kit (Zymo Research Corporation, Irvine, CA, USA) to remove melanin pigment residuals. Samples were quantified by ND-1000 spectrophotometer (NanoDrop Technologies, Wilmington, DE, USA) and stored at -20°C. Starting from 250 ng, DNA samples were prepared for whole-genome SNP profiling using the GeneChip® Human Mapping 250K Nsp Assay kit (Affymetrix, Santa Clara, CA, USA) and hybridized onto GeneChip® Human Mapping 250K Nsp Arrays (Affymetrix), according to manufacturer's instructions. Chips were washed and stained on the Fluidics Station FS-450 (Affymetrix) and scanned by the GeneChip® Scanner 3000 7G (Affymetrix). SNP signal intensities were acquired by GCOS software (Affymetrix) to generate raw intensity (.CEL) files and GTYPE BRLMM algorithm (Affymetrix) was used to assign SNP calls. All chips had a global SNP call rate above 95% and were included in the analysis.

**Whole-genome copy number alteration analysis**

Genome-wide analysis of copy number alterations (CNAs) occurring in each melanoma cell line was performed using Partek Genomics Suite software (version 6.5; Partek Inc., St Louis, MO, USA). Starting from .CEL files, SNP signal intensities were processed by using Partek algorithm for normalization, summarization and quantification, with default parameters (pre-background normalization adjustment for probe sequence and fragment length and allele-specific summarization). Then, to convert signal intensities into SNP copy number (CN) values, a common reference pool including 270 HapMap normal samples profiled by the same GeneChip® 250K SNP Array platform and included in Partek software was used as baseline for each sample. To identify statistically significant CNA regions in each melanoma cell line, the "genomic segmentation" algorithm was applied, setting 10 contiguous SNPs as minimum number of markers, $p$-value $< 0.001$, signal to noise ratio at 0.3 and False Discovery Rate (FDR) $< 0.05$. Only autosomal chromosomes (from 1 to 22) were considered for further analyses. Concerning deleted regions, we assumed mean CN value less than 1.3 as indication of one-copy loss (heterozygous deletion) and mean CN value less than 0.9 as two-copy loss (homozygous deletion, HD).

**Whole-exome capture and sequencing**

Whole-exome capture and library preparation was performed using the Agilent SureSelect$^{XT}$ Human All Exon 50Mb kit (Agilent Technologies, Santa Clara, CA, USA), according to the SureSelect$^{XT}$ Target Enrichment System for Illumina Paired-End Sequencing Library protocol (version 1.1.1; Agilent Technologies). Briefly, 3 ug of genomic DNA per sample were sheared using a Covaris S2 AFA instrument (Covaris, Woburn, MA, USA) to a target peak size of 150-200 bp. After fragmentation check by Agilent 2100 BioAnalyzer microcapillary electrophoresis (Agilent Technologies), the exome capture protocol was performed according to manufacturer's instructions and included genomic DNA library preparation and then target enrichment by using Agilent SureSelect exome capture baits. Finally, post-capture libraries were loaded, one sample per lane, onto the Illumina cBot Cluster Generation System (Illumina, San Diego, CA, USA) at a 8 pM final concentration, and then sequenced by Illumina GAIIx instrument, in a paired-end 76-cycle run. For raw data processing, the Sequencing Control Software (SCS, Illumina) was used to convert raw image data into qseq files and then CASAVA software (Illumina) to generate final fastq files.

**Whole-exome sequencing data analysis**

Whole-exome sequencing (WES) paired-end reads were mapped to the NCBI human reference genome GRCh37 build using MAQ aligner (http://maq.sourceforge.net/) with default parameters. All the reads with more than 5 mismatches and mapping quality (MAPQ) less than 10 were filtered out, using the *submap* option of MAQ package. After duplicate removal, the native mapping files (.map) were converted into the .bam standard files using the *maq2sam-long* tool in SAMtools package (v.0.1.12a) (Li et al., 2009). Here, we focused our attention on single nucleotide variants (SNVs), without addressing further investigations to small insertions/deletions (indels) or other possible structural variations. Thus, .bam files were used for SNV analysis using GATK software (v.1.0.5506) (DePristo et al., 2011), with the following procedure: (a) reads were recalibrated using *TableRecalibration* walker to bring quality scores closer to their actual probability of mismatching the reference genome; (b) reads were realigned around known indels using *IndelRealigner* walker and the 1000 Genomes Project data (http://www.1000genomes.org) to improve the alignment quality; (c) the *UnifiedGenotyper* walker was used to perform SNV calling, thus generating a VCF file containing all the raw variations detected for each sample; (d) groups of three SNVs within 10 base-windows were flagged as "SNPCluster" in the FILTER field of the VCF file.

Then, a series of filtering options was applied to create a sub-selection of high-quality SNVs (HQ-SNVs) for further analyses (Suppl Figure S1). The filtering criteria were: (i) SNV position was covered by at least 15 reads (i.e. minimum read depth of 15x); (ii) SNV call quality score (as reported in the QUAL field of the VCF file) was greater than 150; (iii) allele frequency of non-reference variant was above 20%; (iv) SNV mapped in coding regions; (v) SNV was not already reported as known polymorphism in NCBI dbSNP132 database (including also Phase I 1000 Genomes Project variants), except for the SNVs annotated as "clinically relevant" which were maintained in our dataset; (vi) SNVs mapping on HLA loci were filtered out, as well as (vii) those mapping on homologous repeated genes according to the Duplicated Genes Database (v61, http://dgd.geneoust.org); (viii) SNV positions overlapping to variants already found in our in-house database of WES projects were disregarded to exclude technical or bioinformatics systematic errors. Thus, by applying these filtering options on VCF files, we finally generated a dataset of novel coding HQ-SNVs, including both synonymous and non-synonymous variants (introducing amino acid change (missense) or stop codon (nonsense)). On the basis of the ratio calculated by

GATK between non-reference and reference reads (termed "allele frequency" (AF)), SNVs were classified as heterozygous (AF=0.5) or homozygous (AF=1) variants. Finally, SIFT tool was used to predict potential protein-function damage for non-synonymous missense HQ-SNVs (http://sift.jcvi.org/).

**Concordance with SNP array genotype calls**

To assess reliability and sensitivity of our WES data for SNV detection, genotype calls generated from WES and Affymetrix SNP array for the six samples were compared and their concordance was evaluated in relation to sequencing read depth. First, the genomic coordinates of all the SNPs interrogated by the Affymetrix GeneChip® Human Mapping 250K SNP Array (originally annotated on UCSC hg18 release) were converted into their hg19 equivalents by using UCSC *LiftOver* tool (http://genome.ucsc.edu/cgi-bin/hgLiftOver). Also, for each SNP, the Affymetrix genotype calls were converted from categorical (AA, AB or BB) to allele frequence (AF)-like values, considering the hg19 nucleotide base as reference. So, AF=0 was assigned to wild-type homozygous positions, AF=0.5 to heterozygous positions, and AF=1 to non-reference homozygous positions. Regarding WES data, we retrieved the reads for all the genomic positions corresponding to the GeneChip® 250K SNPs and having at least a 2x coverage. To work with a maximally informative set of genotype calls, we considered only the SNP positions consistently covered across all the six samples, for a total of about 6,500 informative SNPs. WES genotypes were categorized according to the following criteria: a) all positions with a ratio of non-reference/reference reads below 0.2 were considered homozygous wild-type (AF=0); b) all positions with a ratio between 0.2 and 0.8 were considered heterozygous variations (AF=0.5); c) all positions with a ratio above 0.8 were considered homozygous non-reference variations (AF=1). For each category, we determined the concordance (defined as the ratio of concordant classifications on the total number of variations) between the two platforms in relation to the sequencing read depth.

**Bioinformatics analysis**

***COSMIC gene list***

SNV and CNA data were compared with Sanger Catalogue Of Somatic Mutations In Cancer (COSMIC database v58, ftp://ftp.sanger.ac.uk/pub/CGP/cosmic/) to characterize our samples with respect to this comprehensive cancer gene database. To assess mutation overlap, we considered the perfect match on punctual genomic positions between COSMIC mutations and our non-

synonymous HQ-SNVs. To evaluate deletion overlap, we started from genes reported in COSMIC as affected by wide deletions spanning the entire locus (so excluding small internal deletions) and considered whether we found a corresponding whole-gene loss in our dataset.

***Manually curated list of melanoma-related genes***

We manually compiled a comprehensive and updated list of melanoma-related genes. Starting from genes already known to be involved in melanoma biology according to traditional knowledge and those proposed as novel candidate genes by recently published literature, we extended the list so to include all the members belonging to those given gene families. Finally, we obtained a list comprising 30 gene families from the canonical KEGG Melanoma pathway (map05218; http://www.genome.jp/dbget-bin/www_bget?path:map05218) and other 26 gene families derived from recent melanoma WES or targeted sequencing papers (Berger et al., 2012; Nikolaev et al., 2011; Prickett et al., 2009; Prickett et al., 2011; Solomon et al., 2008; Stark et al., 2011; Walia et al., 2012; Wei et al., 2010; Wei et al., 2011a; Wei et al., 2011b), for a total of 547 potentially melanoma-related genes (Suppl File S1). We used this list to extensively screen our melanoma cell lines for known and novel mutations in all the genes that, at current knowledge, might have some involvements in melanoma biology. Finally, to highlight particularly impacted molecular processes, mutated genes were grouped according to the signaling cascade or molecular function they belong to.

**Web resources**

All the synonymous and non-synonymous HQ-SNVs found in each melanoma cell line were collected into a SQLite-based public database and are available for free consultation and download in our Melanoma Exome Database (MExDB) at https://155.253.6.64/MExDB/.

Moreover, we implemented a Generic Genome Browser (GBrowse) genome viewer using the Generic Model Organism Database (GMOD) tools (http://gmod.org). The melanoma exome GBrowse is available at http://155.253.6.64/cgi-bin/gb2/gbrowse/melanoma_exome_agilent/ and includes interactive web pages to browse throughout chromosomes and samples, for visualizing read alignments, SNV positions and sequencing read depth and Partek CNA regions.

## REFERENCES


Berger, M. F., Hodis, E., Heffernan, T. P., Deribe, Y. L., Lawrence, M. S., Protopopov, A., Ivanova, E., Watson, I. R., Nickerson, E., Ghosh, P., et al. (2012). Melanoma genome sequencing reveals frequent PREX2 mutations. Nature *485*, 502-6.

Depristo, M. A., Banks, E., Poplin, R., Garimella, K. V., Maguire, J. R., Hartl, C., Philippakis, A. A., Del Angel, G., Rivas, M. A., Hanna, M., et al. (2011). A framework for variation discovery and genotyping using next-generation DNA sequencing data. Nat Genet *43*, 491-8.

Li, H., Handsaker, B., Wysoker, A., Fennell, T., Ruan, J., Homer, N., Marth, G., Abecasis, G., and Durbin, R. (2009). The Sequence Alignment/Map format and SAMtools. Bioinformatics *25*, 2078-9.

Nikolaev, S. I., Rimoldi, D., Iseli, C., Valsesia, A., Robyr, D., Gehrig, C., Harshman, K., Guipponi, M., Bukach, O., Zoete, V., et al. (2011). Exome sequencing identifies recurrent somatic MAP2K1 and MAP2K2 mutations in melanoma. Nat Genet *44*, 133-9.

Prickett, T. D., Agrawal, N. S., Wei, X., Yates, K. E., Lin, J. C., Wunderlich, J. R., Cronin, J. C., Cruz, P., Rosenberg, S. A., and Samuels, Y. (2009). Analysis of the tyrosine kinome in melanoma reveals recurrent mutations in ERBB4. Nat Genet *41*, 1127-32.

Prickett, T. D., Wei, X., Cardenas-Navia, I., Teer, J. K., Lin, J. C., Walia, V., Gartner, J., Jiang, J., Cherukuri, P. F., Molinolo, A., et al. (2011). Exon capture analysis of G protein-coupled receptors identifies activating mutations in GRM3 in melanoma. Nat Genet *43*, 1119-26.

Solomon, D. A., Kim, J. S., Cronin, J. C., Sibenaller, Z., Ryken, T., Rosenberg, S. A., Ressom, H., Jean, W., Bigner, D., Yan, H., et al. (2008). Mutational inactivation of PTPRD in glioblastoma multiforme and malignant melanoma. Cancer Res *68*, 10300-6.

Stark, M. S., Woods, S. L., Gartside, M. G., Bonazzi, V. F., Dutton-Regester, K., Aoude, L. G., Chow, D., Sereduk, C., Niemi, N. M., Tang, N., et al. (2011). Frequent somatic mutations in MAP3K5 and MAP3K9 in metastatic melanoma identified by exome sequencing. Nat Genet *44*, 165-9.

Villablanca, E. J., Raccosta, L., Zhou, D., Fontana, R., Maggioni, D., Negro, A., Sanvito, F., Ponzoni, M., Valentinis, B., Bregni, M., et al. (2010). Tumor-mediated liver X receptor-alpha activation inhibits CC chemokine receptor-7 expression on dendritic cells and dampens antitumor responses. Nat Med *16*, 98-105.

Walia, V., Mu, E. W., Lin, J. C., and Samuels, Y. (2012). Delving into somatic variation in sporadic melanoma. Pigment Cell Melanoma Res *25*, 155-70.

Wei, X., Prickett, T. D., Viloria, C. G., Molinolo, A., Lin, J. C., Cardenas-Navia, I., Cruz, P., Rosenberg, S. A., Davies, M. A., Gershenwald, J. E., et al. (2010). Mutational and functional analysis reveals ADAMTS18 metalloproteinase as a novel driver in melanoma. Mol Cancer Res *8*, 1513-25.

Wei, X., Moncada-Pazos, A., Cal, S., Soria-Valles, C., Gartner, J., Rudloff, U., Lin, J. C., Rosenberg, S. A., Lopez-Otin, C., and Samuels, Y. (2011a). Analysis of the disintegrin-metalloproteinases family reveals ADAM29 and ADAM7 are often mutated in melanoma. Hum Mutat *32*, E2148-75.

Wei, X., Walia, V., Lin, J. C., Teer, J. K., Prickett, T. D., Gartner, J., Davis, S., Stemke-Hale, K., Davies, M. A., Gershenwald, J. E., et al. (2011b). Exome sequencing identifies GRIN2A as frequently mutated in melanoma. Nat Genet *43*, 442-6.
